# Advice Wanted

**Published:** 1891-06

**Authors:** A. T. Noe

**Affiliations:** Bethany Heights, Lincoln, Neb.


### ADVICE WANTED.

A. T. Noe, M. D., Bethany Heights, Lincoln, Neb.

Mrs. L. F., age twenty-four years, tall, dark hair and eyes and complexion; bilious temperament; weight one hundred and thirty-five to one hundred and forty pounds. Has been married five years; has two children; both are girls. The older one is four years, and the younger one two years old. One miscarriage about a year ago, caused by "La Grippe." She is very jealous. If her husband speaks to a certain lady, she gets angry, cries, and threatens to shoot herself or the one she hates. She does not hate her husband at all. She has no *reason* to be jealous of him. There never was a better man than her husband; but she has become suspicious of a certain lady, and gets angry every time she sees her or thinks about her. She imagines that the woman is trying to break up or disturb her domestic relations, or ruin them as she often expresses it.

I have seen her sitting in church intently watching her enemy during the whole service. She has been heard to say that she went to church purposely to watch her husband, yet it is certain that she has no rational grounds for her jealousy. I have known her to kiss her children farewell, give them her rings and other jewelry, and leave the house with a revolver, threatening to shoot herself. When her husband interfered, she would take the butcher-knife and, offering it to him, beg him to kill her. When he refused, she would threaten to take poison. She has been acting in this manner ever since her first baby was born. She assures her husband that she loves him and does not wish to be in his way ; that she cannot stand it and does not want to see him anxious about her conduct, which she declares she cannot help.

She has some leucorrhœa, but very thin and watery. It comes after these spells. She has headache, but mostly on left side. She cannot stand much noise. Children irritate her. She quickly becomes impatient and sharply threatens them with punishment. Her manner is rough and abusive. Her menstruation is normal.

Every spring, as soon as warm weather comes on, her feet become painful, with cramp in the soles. Her corns are sore and painful, with some stinging and burning in them. She is of a cold nature. Can't stand being left alone. She thinks and studies about her husband all the time he is gone, and is happy only when he is at home.

Will the readers of THE HOMŒOPATHIC PHYSICIAN give me some suggestions as to the remedy indicated in this case?

